# Electrophysiological Insights in Exergaming—Electroencephalography Data Recording and Movement Artifact Detection: Systematic Review

**DOI:** 10.2196/50992

**Published:** 2025-04-07

**Authors:** Carolina Rico-Olarte, Diego M Lopez, Bjoern M Eskofier, Linda Becker

**Affiliations:** 1 Telematics Department Universidad del Cauca Popayán Colombia; 2 Machine Learning and Data Analytics Lab Friedrich-Alexander-Universität Erlangen-Nürnberg Erlangen Germany; 3 Department of Psychology Friedrich-Alexander-Universität Erlangen-Nürnberg Erlangen Germany

**Keywords:** exergaming, EEG, brain activity, motion artifact, artifact removal

## Abstract

**Background:**

Exergames are interactive solutions that require physical activity and are commonly used in learning or rehabilitation settings. For cognitive rehabilitation with exergames, the assessment of the intervention progress can be conducted by verifying the changes in brain activity. Electroencephalography (EEG) is a well-known method for this evaluation. However, motion artifacts due to large body movements can impede signal quality. No comprehensive guide on the artifact removal methods in the context of exergaming has been found.

**Objective:**

This paper aimed to identify studies that have assessed EEG signals while a user interacts with an exergame and the applied methods for data handling and analysis with a focus on dealing with movement artifacts.

**Methods:**

This review included studies on human participants while engaging in exergames, where the primary outcome was brain activity measured by EEG. A total of 5 databases were searched at 3 time points: March 2021, October 2022, and February 2024. The Quality Assessment Tool for Observational Cohort and Cross-Sectional Studies assessed methodological quality, rating studies as “good,” “fair,” or “poor.” Data were synthesized quantitatively to identify characteristics across studies, including sample demographics and intervention details, and basic statistics (mean [SD]) were calculated.

**Results:**

A total of 494 papers were screened, resulting in 17 studies having been included. All studies carried out EEG recordings during exergame interactions, primarily assessing attention and concentration, with the alpha wave being the most analyzed EEG band. Common motion artifact removal methods included visual inspection and independent component analysis. The review identified significant risks of bias, with 2 studies rated as “good,” 7 as “fair,” and 8 as “poor.” Due to the small number of studies and their heterogeneity, a meta-analysis was not feasible.

**Conclusions:**

The study successfully identifies the feasibility of recording electrophysiological brain activity during exergaming and provides insights into EEG devices, analysis methods, and exergaming systems used in previous studies. However, limitations, such as the lack of sufficient detail on motion artifact removal and a focus on short-term effects, underscore the need for improved methodologies and reporting standards, with recommendations for enhancing reliability in cognitive rehabilitation with exergames.

## Introduction

### Rationale

Over the last 15 years, research around the concept of exergaming has expanded in the scientific community as well as in the commercial industry, such as the development of motion controllers for gaming consoles and virtual reality (VR) headsets [1-4]. The closest to a formal definition is found in [5], where the term exergame refers to “digital games that require bodily movements to play, stimulating an active gaming experience to function as a form of physical activity.” Based on this, the scope of exergames can also extend to include VR, augmented reality, and similar technologies that provide immersive gaming experiences. Due to their nature, exergames are used across interdisciplinary fields, including application areas such as psychology, sports science, neuroscience, and computer science. Indeed, the largest application area is in public health and rehabilitation [6-8].

The main populations targeted for the use of exergames are children and adolescents due to their interest in video games and their potential benefits [9]. Some advantages of exergames are their ability to increase motivation and engagement in physical activity [5,10,11]. They also present features such as individualization, adaptivity (by providing feedback for adjustments), and specificity (task-centered and outcome-specific training) [12]. Consequently, exergaming can have a positive impact on physical, cognitive, and psychosocial variables in users [13].

Given their potential to improve health, exergames have evolved and have been evaluated in several scenarios as promising rehabilitation tools [14-16], especially for cognitive rehabilitation [17,18]. The constant training generated through the interaction with exergames may result in the acquisition or improvement of cognitive abilities such as executive functions (eg, attention and memory) [19]. These cognitive skills are necessary for effective performance of everyday tasks, especially in the case of older adults, as well as for school performance in children [20,21].

One specific exergame, HapHop-Physio, was designed and developed by our research group as part of an ongoing research project. This exergame targets children and adolescents with specific learning disorders, aiming to improve their memory and attention through physical activity. Initial findings are promising in supporting children’s cognitive development and thus, improving their daily routines [22]. In previous studies, electrodermal activity has been recorded during interactions with HapHop-Physio, providing valuable insights into the cognitive processes influenced by exergaming [23].

However, to fully assess cognitive-specific outcomes, exergames like HapHop-Physio require the additional measurement of neurophysiological changes related to brain activity. Our research focuses on evaluating HapHop-Physio’s effectiveness through electroencephalography (EEG) [22]. EEG is one of the most commonly used methods for inspecting electrical activity in the brain by recording brain signals with electrodes placed on the scalp [24,25]. EEG is increasingly used in studies on infant development [23]. EEG captures voltage fluctuations resulting from ionic currents within neurons, which produces signals that represent various brain waves. EEG analysis allows an assessment of the underlying processes of cognitive functions [26]. However, EEG signals are highly sensitive to external noise and interference, such as muscle movement, eye blinks, or electrical noise, which introduces artifacts in the EEG signal (ie, unwanted additional components that distort the raw signal) [27]. These artifacts pose a challenge to the measurement of brain activity during the interaction with an exergame, since movements cannot be avoided during exergaming. The movement artifacts complicate or impede the accurate interpretation of the EEG signals. Thus, removing or minimizing artifacts is crucial in ensuring valid EEG readings [28].

Consequently, some researchers have addressed this issue from different approaches. Artifact removal methods vary from manual visual inspection or the application of band filters to complex procedures such as independent component analysis (ICA) or regression-based techniques. ICA aids in detecting facial muscle movements, eye blinks, or other movements. Regression-based techniques predict and subtract the contribution of artifacts to the signal (particularly from eye movements) using mathematical models [29-31]. The specific choice of artifact removal method depends on the specific study design, the types of artifacts present, and the trade-offs between preserving brain signals and removing noise [32].

Nevertheless, the body movements generated by the interaction with an exergame are a piece of motion artifact that are just beginning to be explored. Accurately quantifying data loss due to artifacts is important because large portions of EEG data may be discarded, leading to reduced sample sizes or biased results. When reporting data loss, the research community benefits from better interpretation and an effective comparison across studies. Evaluating signal quality becomes essential to determine whether the brain activity signals remain useful for interpretation after cleaning [33]. Similarly, the associations between any specific cognitive domain induced by the interaction with the exergame and its content are not yet generalizable and depend on the type of exergame as well as the type and strength of body movements.

### Objectives

The primary objective of this systematic review was to identify studies that have measured EEG signals during exergame interaction with a focus on examining the methods used for artifact detection and removal addressing the “large” movement artifact generated. Thus, this review aimed to answer the following questions:

1. Main review questions: (1) Can electrophysiological brain activity be recorded during exergaming? (2) Which EEG devices, methods for data analysis with a focus on methods for artifact detection and removal, and exergaming systems have been used in previous studies?
2. Further review questions: (1) Which population groups have been studied so far (eg, with respect to health status, age, gender, ethnicity, and education)? (2) Are there differences in brain activity during exergaming across the lifespan (eg, between children, adolescents, younger adults, and older adults)?

## Methods

### Eligibility Criteria

The PRISMA (Preferred Reporting Items for Systematic Reviews and Meta-Analyses) statement [34] was followed to report the process and results obtained in the revision (Multimedia Appendix 1). In this systematic review, we established explicit inclusion and exclusion criteria to determine the eligibility of studies. Detailed specifications of these criteria are provided in Textbox 1.

**Details on eligibility criteria.**

**Inclusion criteria**

- Human participants, all age groups.
- All types of exergames and cognitive-motor games.
- Original studies, longitudinal or experimental studies, (controlled) cross-sectional studies, or case-control studies.
- Electrophysiological activity (via electroencephalography [EEG]), participants’ characteristics (eg, age, gender, and health status), characteristics of the game and the EEG device, and the preprocessing pipeline.
- Brain activity measured by EEG during the exergame activity.

**Exclusion criteria**

- Animal studies.
- Single cognitive or motor tasks (eg, classical video games).
- Brain-computer interface games.
- Literature reviews, case reports, and qualitative studies.
- Other neuroscientific methods (eg, functional near-infrared spectroscopy, magnetoencephalography, and functional magnetic resonance imaging).

### Information Sources

We used 5 bibliographic databases to identify peer-reviewed studies for their inclusion in this systematic review: PubMed, IEEE Xplore, ACM Digital Library, Scopus, and Web of Science. Also, we searched the reference list from retrieved full-text articles for additional studies. To keep the results up to date, we conducted searches based on 2 search strings at 3 separate times. The first search date was March 2021, the second search date was October 2022; and finally, the third search was performed in February 2024.

### Search Strategy

For the search strategy, we considered 2 main concepts to retrieve the studies: “exergaming” and “electroencephalography.” We adjusted these concepts according to the respective databases’ Thesaurus and MeSH (Medical Subject Headings) terms. Also, a second search string was built to replace the concept of exergaming with commercial devices that meet the concept. Thus, for the first string, we searched 10 synonyms within the exergaming concept and 36 synonyms within the EEG concept covering the obtained measures when recording brain activity through EEG; as for the second string, we searched for eleven devices (mostly VR headsets) along with the electroencephalography concept. Multimedia Appendix 2 describes the strings, filters, and limits used in each information source. A further source of information was the references to the papers that supplied the following selection process.

### Selection Process

After retrieving the studies from the information sources, we uploaded the files to the Rayyan platform [35] to remove the duplicates and start the screening and selection process. We established a 3-stage approach. First, 2 authors (CRO and LB) independently screened all the titles and abstracts to identify articles that potentially met the inclusion criteria. Second, the introduction and conclusions of the remaining articles were screened to further refine the selection. Finally, full texts of the articles were independently assessed for eligibility and data extraction. Any disagreements between the 2 raters were solved through discussion with a third author (DML). The Rayyan platform enabled blinded screening and selection of papers.

### Data Collection Process

We extracted data from the selected papers generating a standardized prepiloted spreadsheet. As in the selection process, 2 authors (CRO and LB) collected the data independently. The spreadsheet comprised study identification, methodological design, sample characteristics, details of the exergame and the EEG device, analysis methods, outcomes, study limitations, and some additional information.

### Data Items

The most relevant conditions for the papers to be selected were (1) that there was an exergame, that is, a game that stimulates the participants both physically and cognitively (or generates entertainment); (2) that authors recorded brain (EEG) activity during the game, that is, while the participants were stimulated and were moving; (3) that authors provided a detailed description of the EEG analysis methods.

Other variables considered included whether the application was for clinical patients, the data collection setting (laboratory, clinic, or at home), characteristics of the intervention, results in terms of cognition and brain activity, and study limitations; and covariates.

### Study Risk of Bias Assessment

The 2 authors (CRO and LB) independently evaluated the methodological quality of the selected studies. The Quality Assessment Tool for Observational Cohort and Cross-Sectional Studies [36] from the National Heart, Lung, and Blood Institute (NHLBI) [37] contains fourteen questions designed to assess the internal validity of a study. Any disagreement in the evaluation was solved by the third author (DML).

The evaluation process consisted of 2 steps. First, we assigned importance levels to the 14 yes or no questions based on the elements queried by each question, such as research question, sample size justification, etc. Four questions were classified as very important, 1 as important, and 9 as less important. The importance level determined the weight of each question in the assessment process.

Subsequently, we evaluated each study based on its responses to the yes or no questions. A “good” rating was assigned when none or just 1 answer was “NO,” indicating minimal risk of bias. A “fair” rating was given when 2 or 3 answers were “NO,” suggesting some potential for bias. Conversely, a “poor” rating was assigned when there was a “NO” answer in 1 of the most important questions, or at least 4 “NO” answers in the less important questions, indicating a significant risk of bias. The final rating for each study was determined by tallying the number of “NO” answers across all questions.

### Synthesis Methods

From the data collected and recorded in the matrix, we synthesized evidence quantitatively to determine the sets of characteristics that could be associated with each study, such as the population sample, the countries that conducted the research, or the gender distribution of the sample. In the same way, we calculated basic statistics (mean [SD]) for characteristics such as the ages reported in the studies and the intervention performed with the exergame and measured with the EEG device. A meta-analysis should be considered depending upon the availability of appropriate data (ie, sufficient number and homogeneity of studies).

The protocol for this systematic review was prospectively registered in the PROSPERO (International prospective register of systematic reviews) database (registration no CRD42020208131). No deviations from the registered protocol occurred during the review process.

## Results

### Study Selection

**Figure 1:**
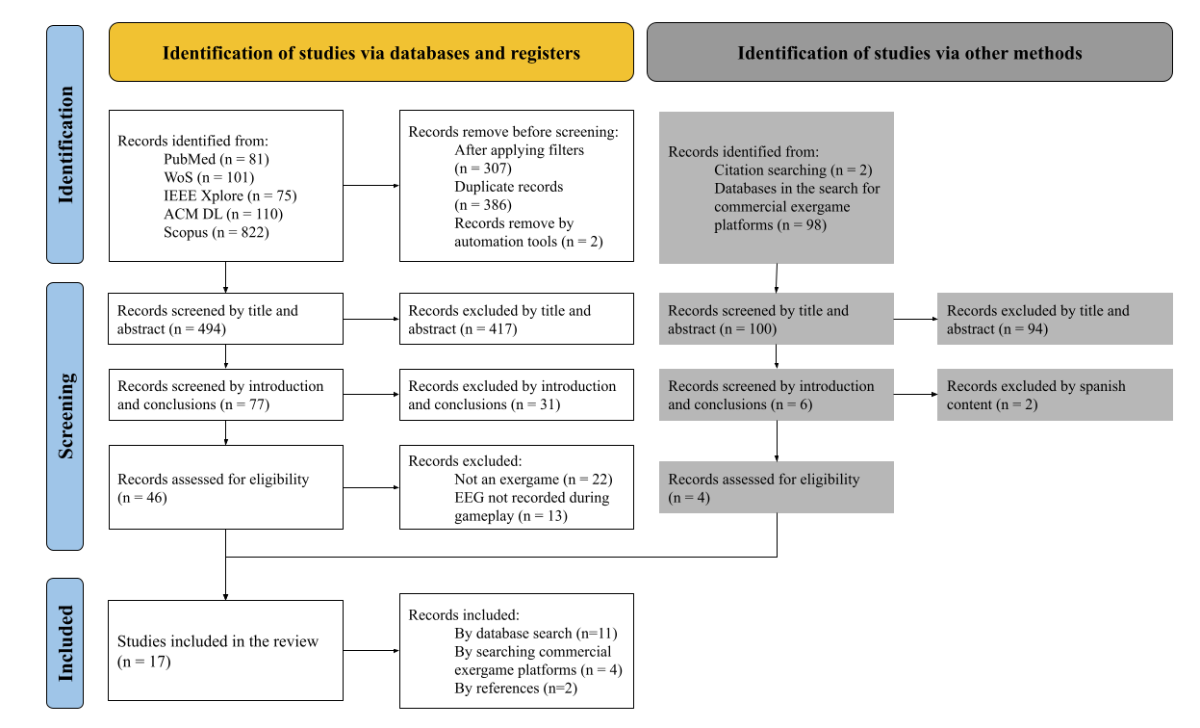
Flowchart of the results from the review process.

After removing the duplicates, we first screened 494 papers by title and abstract. Second, we screened 77 papers by introduction and conclusions. Third, we reviewed the full text of 46 documents, excluding 35 studies from this set, either because they did not refer to an exergame (n=22), or did not record EEG activity during gameplay (n=13; Figure 1). Finally, only 11 papers were included in the review. Additional papers fulfilling the inclusion criteria were found in other searches, such as the study’s references (n=2) and the search for commercial exergames platforms (Nintendo Wii and Kinect) that were not included in the searches results since they were referenced by their commercial name and not as exergames (n=4).

A considerable number of papers containing the keywords were excluded because they were within the brain-computer interface (BCI) paradigm. This paradigm uses EEG as an input device to control the game from the brain activity detected in the player. Although papers reported EEG recordings, there was no relationship reported between the physical activity generated by the game (not classified as exergame) and the brain signals.

### Study Characteristics

The 17 included studies were conducted in diverse continent settings including Europe (5 studies: Norway, Germany [n=3] Austria, and Italy); Asia (5 studies: Korea, China [n=2], Turkey, and Iran); North America (3 studies: the United States and Canada [n=2]), Central America (1 study from Mexico), South America (2 studies from Brazil) and 1 in Oceania (New Zealand). Sample sizes across these studies were generally small, with 6 studies including between 10 and 19 participants, and 4 studies reporting more than 30 participants. Authors of 11 of the identified studies labelled the design as “experimental.” The remaining studies were reported to be pilot studies (n=2), a feasibility study (n=1), a preliminary study (n=1), a comparative study (n=1), and a case study (n=1).

In terms of population characteristics, 13 studies reported gender distribution. The female participation rate was 42% against a male participation rate with 58% (mean values from studies). In total, 16 studies reported the age of the participants. The mean age of the study’s participants within the young adult category (n=12) was 25.4 years. The population in 14 of the studies was nonclinical; 2 studies reported data from a clinical population (upper limb injuries and stroke). The children’s population was mixed between clinical (diagnosed with Autism) and a nonclinical sample.

Multimedia Appendix 3 [38-54] shows the characteristics of the included studies. All 17 studies used strategies to induce physical activity: 7 of them classified their games as exergames, 3 studies used the Kinect device, a motion-sensing input device developed by Microsoft for the Xbox gaming console that detects physical movements and translates them into game controls, to detect movement, 3 studies used the Nintendo Wii device, a gaming console with motion-sensing controllers that track physical movements to control game actions, to induce movement, 2 studies use a VR-based approach (MetaQuest2 and HTC Vive), 1 study used the SilverFit system, a rehabilitation device designed for motor and cognitive therapy that combines interactive gaming with physical exercises, and the remaining study used a static bike to induce the physical activity while playing the video games.

Multimedia Appendix 4 [38-60] details the information of EEG recordings in the studies. A total of 12 studies reported methods for the detection and removal of motion artifacts in the EEG signal. There were 2 studies for which the process is not transparent since it is made internally by the devices. The EEG activity was measured by its band waves (alpha, beta, theta, gamma, and delta) in 14 studies, 2 studies reported the brain activity in terms of the signal components (such as the N1 component of the Event-Related Potential) and finally, 1 study reported the processed signal from the device’s software. A narrative description of the results of individual studies is reported in Multimedia Appendix 5.

### Synthesis of Results

According to the results obtained and from the data extracted from the reviewed papers, the following aspects can be synthesized.

#### Exergame Characteristics

Regarding the exergames used in the studies, 11 out of 17 exergames are commercially available; only 6 exergames were developed to assess the research objectives outlined in each paper. The additional tools that authors used to induce physical activity within the exergame correspond first to platforms for users to stand with different modalities (to apply pressure, to control balance, or to practice specific ski movements), followed by, the motion detection of body gestures and positions through the Kinect and Wii, and finally, VR headset devices.

#### Cognitive Domains and EEG Analysis

The cognitive domains stimulated by the exergames and identified through the analysis of the EEG recordings include attention and concentration (n=4), followed by engagement (n=2), cognitive workload (n=2), stress (n=2), motor learning (n=2), working memory (n=1), and awareness (n=1). Only 1 study evaluated processing speed and language. Two studies did not report the cognitive domains.

In the studies using the analysis of the EEG recordings through band waves, the alpha wave was the most often analyzed (n=10), followed by the beta and theta waves (n=7). The least used waves were gamma and delta. Authors used various methods to measure their results according to the EEG band waves. Among them, the absolute power and the relative power were obtained, in addition to the asymmetrical comparisons between channels (in dB), changes in the bands (in terms of percentages), and applying equations for different indexes based on the values of the bands.

The most common electrode placement positions for EEG recording were the frontal and parietal lobes (n=6), followed by the prefrontal lobe (n=5), then, the central and precentral lobes (n=3). The least used positions were located occipitally (n=2) and temporally (n=1).

#### Motion Artifact Handling

Finally, in line with the aim of this systematic review, the methods for detecting and removing motion artifacts were summarized. The most frequently reported methods were filtering the signal (n=6), using the ICA method (n=4), visual and manual inspection (n=2), and applying the automated subspace reconstruction method (n=2). Other methods used by the authors were based on previous contributions such as plug-ins, pipelines, and metrics, including the use of several types of band-pass filters. Some authors used recording devices that automatically filtered motion artifacts by deleting them before delivering the data to the user.

### Risk of Bias Assessment in Studies

The risk of bias was evaluated across 2 dimensions: internal validity (risk of bias within individual studies) and external validity (reporting biases affecting the overall synthesis).

The methodological quality of the studies included was assessed based on essential factors like study population, exposure measures, and statistical analysis. Among the studies, only 2 [40,50] were rated as “good,” indicating the least risk of bias and validating their results. Seven studies [38,39,45,47,49,51,53] were rated as “fair,” suggesting some potential for bias, though not enough to invalidate their findings. However, 8 studies [41-44,46,48,52,54] received a rating of “poor,” indicating a significant risk of bias. These studies were included in the review due to the limited availability of alternative evidence. Full details of the risk of bias assessment are provided in Multimedia Appendix 6.

In the assessment of reporting biases, 2 papers received a “good” rating, indicating a minimal risk of bias. These papers demonstrated clear objectives, transparent recruitment process, and consistent exposure and outcome measure implementations. Seven papers were rated as “fair” suggesting a moderate risk of bias, due to shortcomings such as insufficient sample size justification or limited exposure assessment at only 1 time point. In contrast, the remaining 8 papers were rated as “poor” indicating a high risk of bias. These papers lacked clear definition of the study population, exhibited inconsistent implementation of outcome measures, and had an unclear recruitment process. In addition, none of the papers reported blinding of assessors to participant exposure status, and none accounted for confounding variables. Furthermore, some papers included unnecessary information in their reporting.

### Certainty of Evidence

Unfortunately, the number of papers that met the inclusion criteria to fulfill the aim of this systematic review was low (n=17). This low number of studies did not allow for a meta-analysis of the information extracted from each paper due to the heterogeneity of the studies. Likewise, it is not possible to generalize the results, since the risk-of-bias assessment resulted in inferior quality in 47% of the papers analyzed.

Two papers [40], [50] mentioned the need to detect and remove movement artifacts from the EEG signal recorded during the participants’ interaction with the exergame. One important piece of information from [40] was the decision to remove around 50% of the signal. In contrast, for the EEG signal epochs in [50], only between 5% and 7% were removed. In the remaining papers, although noise detection and removal methods were applied, no specific reference was made to the motion artifacts generated by physical activity during playing the exergame or the amount of signal that was removed for further analysis.

## Discussion

### Principal Findings

Our review revealed several key findings regarding the recording of electrophysiological brain activity during exergaming. First, we demonstrated the feasibility of this approach, highlighting the potential for capturing neural signals during interactive physical activity. In addition, we identified a variety of EEG devices, analysis methods, and exergaming systems used in previous studies, providing valuable insights into the technological landscape of the field. The analysis also covered significant challenges, including issues related to data loss and motion artifact removal, which impact statistical power and introduces bias in estimation of EEG outcomes. We observed a lack of reporting of detailed information on motion artifact identification, particularly in relation to the motion artifacts generated by large, whole-body movements inherent to exergame interaction, removal methods in the included studies, traditional techniques were primarily developed to address smaller localized artifacts and may not effectively account for the more “substantial” artifacts generated by whole-body movements. This lack of detail highlights the need for greater methodological transparency in future research, especially in how larger motion artifacts are managed to ensure valid EEG findings.

Our review highlighted a notable gap in literature, with few studies conducting long-term measurements of the cognitive and physical effects of exergaming through EEG recordings. There is a predominant focus on young adult populations in existing research, with limited exploration of differences across age groups and ethnicities. These findings collectively contribute to a deeper understanding of the challenges and opportunities in the field of electrophysiological research in exergaming.

The main review questions (Can electrophysiological brain activity be recorded during exergaming? Which EEG devices, methods for data analysis, and exergaming systems have been used in previous studies?) were answered: at least 1 of the included studies carried out a feasibility study determining that, although approximately 50% of the EEG signal data were lost, it was possible to record and subsequently analyze the information from the signals that were obtained while the players interacted with the exergame. This finding, along with the much lower amount reported in [50], is contradictory and highlights the importance of defining comparable quality metrics for future studies. However, data loss negatively impacts the results in several ways. First, it reduces the statistical power of the study. Second, data loss can introduce bias in the estimation of parameters in classification or prediction models, leading to inaccurate results. Finally, it may diminish the representativeness of the sample, which may limit the generalization of the findings [61].

### Comparison to Previous Work

In addition, information on EEG devices, analysis methods, and exergaming systems was obtained and reported in this review. Of the 16 included studies, although all recorded EEG signals, the aims were different in terms of determining the feasibility or quality of the signal in the presence of movements (physical activity) made by the participants. Of these studies, 12 papers attempted to detail the process of identifying and removing motion artifacts from the signal. However, the information provided is not sufficient for replicating the procedures.

Regarding the devices found in the included studies, we classified them according to the type of recording hardware. Four of the EEG devices can be classified as complete systems; that is, they have a signal amplifier and a cap that facilitates the easy placement of EEG electrodes on the participant’s head. Three of the studies simply used a signal amplifier, without mentioning the type of electrodes used for signal capturing or using generic EEG electrodes. Ten studies used headset-type devices. These devices are wireless and store the signal in their memory, which is then downloaded to a software for reading and analyzing. These devices are wearables [62], so their use is much easier for nonexperts in the placement of conventional electrodes according to the 10-20 reference system [63]. In terms of usability in environments other than clinical, wearable devices are convenient to validate hypotheses before conducting more formal studies [64].

Another aspect that was evaluated within this review was the analysis method for processing the EEG data. Of the studies included, 14 used analysis methods in the frequency domain, obtaining the absolute power of the band waves [65]; this is the most commonly used approach in the literature. Only 1 study included time-frequency maps showing increasing and decreasing power events (event-related desynchronization/ event-related synchronization). Two studies analyzed the signal in the time domain, examining the N1 component and using its amplitude metrics. Defining the analysis methods is an important task to determine the effects of movement on the EEG signal and, at the same time, obtain information on the changes or cognitive processes that are activated during the interaction with an exergame [66].

Exergames are still being tested to corroborate the benefits attributed to them, such as the increase in motivation and enjoyment, the reach of populations, and their capacity for individualization, adaptivity, specificity, and scalability [5]. Even though 9 of the studies included used game systems that were not categorized as exergames by the study’s authors (using a Kinect, a Nintendo Wii, stationary bicycle, a balance board, and a VR headset), the authors attributed improvement of cognitive processes to the physical activity during the games [67,68].

In addition to our systematic review, a recent literature review on motion artifact reduction in BCI systems [69], highlights the challenges associated with EEG data collection during physical activities, which closely mirrors our findings in exergaming studies. Both reviews highlight the limitations of widely used artifact removal techniques, such as ICA and regression-based methods, which are often insufficient when addressing the larger artifacts caused by whole-body movements in exergaming. This aligns with our observation that many exergaming studies lack sufficient detail for replicating effective motion artifact removal techniques.

Furthermore, the studies reviewed in [69] revealed a tendency to focus on introducing new methods for artifact reduction, rather than improving or thoroughly comparing established ones. Both our review and the BCI literature emphasize the difficulty of generalizing findings due to small sample sizes and the variations in experimental paradigms, hardware setups, and preprocessing steps. Furthermore, the “ground truth” problem (distinguishing between true brain signals and noise) identified in [69] is especially relevant in exergaming contexts, where participant movement is integral to the activity. By framing our findings within this broader context, we emphasize the need for future research to improve artifact removal methods, enhance study transparency, and adopt advanced technologies to refine EEG signal quality in exergaming.

### Limitations

The greatest limitation found in the included studies is the lack of detail in the processes of identification and removal of motion artifacts in the EEG signal. This limitation hinders a comprehensive understanding of data quality and may impact the accuracy of findings. Another limitation is the formality of the studies conducted, as their objectives only go as far as measuring the immediate effect of 1 game session on cognitive processes. While other studies have data on long-term outcomes, they did not measure EEG during gameplay [70-73]. To date, no study includes long-term measurement of the cognitive effect or the effect on the person’s physical state by the periodic use of an exergame with EEG recordings. This narrow scope limits the depth of insight into the sustained benefits or potential drawbacks of exergaming interventions.

Several limitations were identified in our systematic review. Many of the included studies were formative in nature and had small sample sizes, which restrict the generalization of the findings. However, we opted for a systematic review to evaluate the feasibility of recording EEG during exergaming and to assess methods for managing motion artifacts. Despite the heterogeneity across, including the variation in EEG devices, data analysis methods, and exergames used, we aimed to provide structured insight into the field.

Third, the absence of information on differences in brain activity across age groups and ethnicities restricts the generalizability of our findings. Without this crucial demographic context, the applicability of our results to diverse populations is compromised, highlighting the need for more inclusive research approaches in future investigations. While we used a comprehensive search strategy and followed the PRISMA guidelines, some studies may have been missed, particularly unpublished or grey literature.

Heterogeneity across studies, the small sample sizes, and the formative nature of many studies made synthesis challenging, both qualitatively and quantitatively. It also limited our ability to draw firm conclusions or identify trends across the studies included.

### Future Directions

The results of this systematic review provide answers to further review questions (Which population groups have been studied so far, eg, with respect to health status, age, gender, ethnicity, education? Are there differences in brain activity during exergaming across the lifespan, eg, between children, adolescents, younger adults, and older adults?). As stated in the results section, most of the included studies targeted young adults and no differences were examined between age groups even when some exergames were intended for children but evaluated in young adults. Unfortunately, none of the studies specified information about differences in brain activity during exergaming across the lifespan or the ethnicity of the population. This lack of detail represents a limitation when applying the results of published investigations to other contexts, such as comparing age groups [74,75] or targeting multiethnic populations [76].

Future research should focus on more standardized methods, larger and more diverse sample sizes, and long-term evaluations of cognitive and physical outcomes through EEG recordings during exergaming interventions.

### Conclusions

The review demonstrates the feasibility of recording electrophysiological brain activity during exergaming and offers valuable insights into the EEG devices, analysis methods, and exergaming systems used in previous research. Our findings indicate that EEG signals can be successfully measured in the midst of physical activity induced by player interaction with exergames, which may be associated with cognitive domains and processes. However, the presence of motion artifacts may lead to the loss of up to 50% of the EEG signal, posing challenges for data analysis and interpretation. Despite these limitations, our study underscores the potential for conducting meaningful analyses with the available data. Moving forward, research should address these limitations by enhancing reporting standards and methodological practices. Specifically, researchers should provide a rationale for EEG device selection, determine the type and intensity of movements during recording, assess appropriate motion artifact detection and removal methods, report details of data loss due to artifacts, evaluate signal quality post artifact removal, and conduct more formal longitudinal experiments to explore the medium and long-term effects of exergaming on cognitive processes and physical well-being.

Furthermore, our review has practical implications for the development of therapeutic interventions, as exemplified by our design of the HapHop-Physio exergame targeting children with specific learning disorders [77]. This exergame has the potential to assist in cognitive rehabilitation for this population [17]. By incorporating EEG signal recording during gameplay, our ongoing study aims to characterize electrical signals corresponding to typical player movements [78]. More information on the protocol for the study can be found in the Open Science Framework [79].

## Appendix

Multimedia Appendix 1 PRISMA (Preferred Reporting Items for Systematic Reviews and Meta-Analyses) 2020 checklist.

Multimedia Appendix 2 Table A. Filters and limits in information sources from search strings.

Multimedia Appendix 3 Table B. Details of intervention or exposure in the studies.

Multimedia Appendix 4 Table C. Details of EEG recordings in the studies.

Multimedia Appendix 5 Details of the results of individual studies.

Multimedia Appendix 6 Quality assessment for papers.

## Abbreviations

BCI: brain-computer interface
EEG: electroencephalography
ICA: independent component analysis
MeSH: Medical Subject Headings
NHLBI: National Heart, Lung, and Blood Institute
PRISMA: Preferred Reporting Items for Systematic Reviews and Meta-Analyses
VR: virtual reality

## Data Availability

The precise sources (extraction matrix, tables, and figures) for each estimate are described in a data spreadsheet. Available in Open Science Framework [79].

## References

[1] Best JR (2013). Exergaming in youth: effects on physical and cognitive health. Z Psychol.

[2] Street TD, Lacey SJ, Langdon RR (2017). Gaming your way to health: a systematic review of exergaming programs to increase health and exercise behaviors in adults. Games Health J.

[3] Williams WM, Ayres CG (2020). Can active video games improve physical activity in adolescents? A review of RCT. Int J Environ Res Public Health.

[4] O'Loughlin EK, Dutczak H, Kakinami L, Consalvo M, McGrath JJ, Barnett TA (2020). Exergaming in youth and young adults: a narrative overview. Games Health J.

[5] Benzing V, Schmidt M (2018). Exergaming for children and adolescents: strengths, weaknesses, opportunities and threats. J Clin Med.

[6] Gao Z, Chen S (2014). Are field-based exergames useful in preventing childhood obesity? A systematic review. Obes Rev.

[7] Barry G, Galna B, Rochester L (2014). The role of exergaming in parkinson's disease rehabilitation: a systematic review of the evidence. J Neuroeng Rehabil.

[8] Taylor MJD, Griffin M (2015). The use of gaming technology for rehabilitation in people with multiple sclerosis. Mult Scler.

[9] Baranowski T (2017). Exergaming: hope for future physical activity? or blight on mankind?. J Sport Health Sci.

[10] Osorio G, Moffat DC, Sykes J (2012). Exergaming, exercise, and gaming: sharing motivations. Games Health J.

[11] Cacciata MC, Stromberg A, Klompstra L, Jaarsma T, Kuriakose M, Lee J, Lombardo D, Evangelista LS (2021). Facilitators and challenges to exergaming. J Cardiovasc Nurs.

[12] Smits-Engelsman B, Vinçon S, Blank R, Quadrado VH, Polatajko H, Wilson PH (2018). Evaluating the evidence for motor-based interventions in developmental coordination disorder: a systematic review and meta-analysis. Res Dev Disabil.

[13] Gao Z (2017). Fight fire with fire? Promoting physical activity and health through active video games. J Sport Health Sci.

[14] Mat Rosly M, Mat Rosly H, Davis Oam GM, Husain R, Hasnan N (2017). Exergaming for individuals with neurological disability: a systematic review. Disabil Rehabil.

[15] Mura G, Carta MG, Sancassiani F, Machado S, Prosperini L (2018). Active exergames to improve cognitive functioning in neurological disabilities: a systematic review and meta-analysis. Eur J Phys Rehabil Med.

[16] Chan KGF, Jiang Y, Choo WT, Ramachandran HJ, Lin Y, Wang W (2022). Effects of exergaming on functional outcomes in people with chronic stroke: a systematic review and meta-analysis. J Adv Nurs.

[17] Rico-Olarte C, Narváez-Muñoz N, López DM, Becker L, Tovar-Ruiz L (2022). Assessing HapHop-Physio: an exer-learning game to support therapies for children with specific learning disorders. Applied Sciences.

[18] Li K, Wang Y, Wu Z, Yao X, Fan Y (2023). Effectiveness of active exergames for improving cognitive function in patients with neurological disabilities: a systematic review and meta-analysis. Games Health J.

[19] Hötting K, Röder B (2013). Beneficial effects of physical exercise on neuroplasticity and cognition. Neurosci Biobehav Rev.

[20] Gunzenhauser C, Nückles M (2021). Training executive functions to improve academic achievement: tackling avenues to far transfer. Front Psychol.

[21] Corbo I, Casagrande M (2022). Higher-level executive functions in healthy elderly and mild cognitive impairment: a systematic review. J Clin Med.

[22] Tost A, Migliorelli C, Bachiller A, Medina-Rivera I, Romero S, García-Cazorla Á, Mañanas MA (2021). Choosing strategies to deal with artifactual EEG data in children with cognitive impairment. Entropy (Basel).

[23] Noreika V, Georgieva S, Wass S, Leong V (2020). 14 challenges and their solutions for conducting social neuroscience and longitudinal EEG research with infants. Infant Behav Dev.

[24] Müller-Putz GR (2020). Electroencephalography. Handb Clin Neurol.

[25] Troller-Renfree SV, Morales S, Leach SC, Bowers ME, Debnath R, Fifer WP, Fox NA, Noble KG (2021). Feasibility of assessing brain activity using mobile, in-home collection of electroencephalography: methods and analysis. Dev Psychobiol.

[26] Lau-Zhu A, Lau MP, McLoughlin G (2019). Mobile EEG in research on neurodevelopmental disorders: opportunities and challenges. Dev Cogn Neurosci.

[27] Reis PMR, Hebenstreit F, Gabsteiger F, von Tscharner V, Lochmann M (2014). Methodological aspects of EEG and body dynamics measurements during motion. Front Hum Neurosci.

[28] Mumtaz W, Rasheed S, Irfan A (2021). Review of challenges associated with the EEG artifact removal methods. Biomedical Signal Processing and Control.

[29] Independent Component Analysis for artifact removal. EEGLAB Wiki.

[30] Islam MK, Rastegarnia A, Yang Z (2016). Methods for artifact detection and removal from scalp EEG: a review. Neurophysiol Clin.

[31] Gorjan D, Gramann K, De Pauw K, Marusic U (2022). Removal of movement-induced EEG artifacts: current state of the art and guidelines. J Neural Eng.

[32] Mannan MMN, Kamran MA, Jeong MY (2018). Identification and removal of physiological artifacts from electroencephalogram signals: a review. IEEE Access.

[33] Jiang X, Bian GB, Tian Z (2019). Removal of artifacts from EEG signals: a review. Sensors (Basel).

[34] Page MJ, McKenzie JE, Bossuyt PM, Boutron I, Hoffmann TC, Mulrow CD, Shamseer L, Tetzlaff JM, Akl EA, Brennan SE, Chou R, Glanville J, Grimshaw JM, Hróbjartsson A, Lalu MM, Li T, Loder EW, Mayo-Wilson E, McDonald S, McGuinness LA, Stewart LA, Thomas J, Tricco AC, Welch VA, Whiting P, Moher D (2021). The PRISMA 2020 statement: an updated guideline for reporting systematic reviews. BMJ.

[35] Ouzzani M, Hammady H, Fedorowicz Z, Elmagarmid A (2016). Rayyan-a web and mobile app for systematic reviews. Syst Rev.

[36] Study quality assessment tools. National Institutes of Health (NIH).

[37] Background: development and use of study quality assessment tools. National Institutes of Health (NIH).

[38] Anders P, Lehmann T, Müller H, Grønvik KB, Skjæret-Maroni N, Baumeister J, Vereijken B (2018). Exergames inherently contain cognitive elements as indicated by cortical processing. Front Behav Neurosci.

[39] Scherer R, Moitzi G, Daly I, Muller-Putz GR (2013). On the use of games for noninvasive EEG-based functional brain mapping. IEEE Trans. Comput. Intell. AI Games.

[40] Ghani U, Signal N, Niazi IK, Taylor D (2021). Efficacy of a single-task ERP measure to evaluate cognitive workload during a novel exergame. Front Hum Neurosci.

[41] Parent M, Albuquerque I, Tiwari A, Cassani R, Gagnon J, Lafond D, Tremblay S, Falk TH (2020). PASS: a multimodal database of physical activity and stress for mobile passive body/ brain-computer interface research. Front Neurosci.

[42] Xu W, Liang HN, Zhang Z, Baghaei N (2020). Studying the effect of display type and viewing perspective on user experience in virtual reality exergames. Games Health J.

[43] Ko J, Jang SW, Lee HT, Yun H, Kim YS (2020). Effects of virtual reality and non-virtual reality exercises on the exercise capacity and concentration of users in a ski exergame: comparative study. JMIR Serious Games.

[44] Elor A, Powell M, Mahmoodi E, Teodorescu M, Kurniawan S (2022). Gaming beyond the novelty effect of immersive virtual reality for physical rehabilitation. IEEE Trans. Games.

[45] Baumeister J, Reinecke K, Cordes M, Lerch C, Weiss M (2010). Brain activity in goal-directed movements in a real compared to a virtual environment using the nintendo wii. Neurosci Lett.

[46] Kandemir H, Kose H (2021). Development of adaptive human–computer interaction games to evaluate attention. Robotica.

[47] Pacheco TBF, Oliveira Rego IA, Campos TF, Cavalcanti FADC (2017). Brain activity during a lower limb functional task in a real and virtual environment: a comparative study. NeuroRehabilitation: An International, Interdisciplinary Journal.

[48] Dang X, Wei R, Li G (2016). An efficient movement and mental classification for children with autism based on motion and EEG features. J Ambient Intell Human Comput.

[49] Fernandes ABGS, de Melo JCP, de Oliveira DC, Cavalcanti FADC, Postolache OA, Passos PJM, Campos TF (2021). Is motor learning of stroke patients in non-immersive virtual environment influenced by laterality of injury? A preliminary study. J Bodyw Mov Ther.

[50] Olyaei G, Khanmohammadi R, Talebian S, Hadian MR, Bagheri H, Najafi M (2022). The effect of exergaming on cognition and brain activity in older adults: a motor- related cortical potential study. Physiol Behav.

[51] Müller H, Baumeister J, Bardal EM, Vereijken B, Skjæret-Maroni N (2023). Exergaming in older adults: the effects of game characteristics on brain activity and physical activity. Front Aging Neurosci.

[52] GomezRomero-Borquez J, Del Puerto-Flores JA, Del-Valle-Soto C (2023). Mapping EEG alpha activity: assessing concentration levels during player experience in virtual reality video games. Future Internet.

[53] Amprimo G, Rechichi I, Ferraris C, Olmo G (2023). Measuring brain activation patterns from raw single-channel EEG during exergaming: a pilot study. Electronics.

[54] Moinnereau MA, Oliveira AA, Falk TH (2022). Instrumenting a virtual reality headset for at-home gamer experience monitoring and behavioural assessment. Front. Virtual Real.

[55] Mullen T CleanLine. NeuroImaging Tools and Resources Collaboratory (NITRC).

[56] Ian Daly I, Pichiorri F, Faller J, Kaiser V, Kreilinger A, Scherer R (2012). What does clean EEG look like.

[57] Bigdely-Shamlo N, Mullen T, Kothe C, Su K, Robbins KA (2015). The PREP pipeline: standardized preprocessing for large-scale EEG analysis. Front Neuroinform.

[58] Pion-Tonachini L, Kreutz-Delgado K, Makeig S (2019). The ICLabel dataset of electroencephalographic (EEG) independent component (IC) features. Data Brief.

[59] Casiez G, Roussel N, Vogel D (2012). 1 € filter: a simple speed-based low-pass filter for noisy input in interactive systems.

[60] Li R, Principe JC (2006). Principe, Blinking artifact removal in cognitive EEG data using ICA, Conf.

[61] Kang H (2013). The prevention and handling of the missing data. Korean J Anesthesiol.

[62] Mihajlovic V, Grundlehner B, Vullers R, Penders J (2015). Wearable, wireless EEG solutions in daily life applications: what are we missing?. IEEE J. Biomed. Health Inform.

[63] Morley A, Hill L, Kaditis A Welcome to the ERS respiratory channel. ERS Respiratory channel.

[64] Casson AJ (2019). Wearable EEG and beyond. Biomed Eng Lett.

[65] Michel CM (2019). High-resolution EEG. Handb. Clin. Neurol.

[66] Chikhi S, Matton N, Blanchet S (2022). EEG power spectral measures of cognitive workload: a meta-analysis. Psychophysiology.

[67] Stanmore E, Stubbs B, Vancampfort D, de Bruin ED, Firth J (2017). The effect of active video games on cognitive functioning in clinical and non-clinical populations: a meta-analysis of randomized controlled trials. Neurosci Biobehav Rev.

[68] Comeras-Chueca C, Marin-Puyalto J, Matute-Llorente A, Vicente-Rodriguez G, Casajus JA, Gonzalez-Aguero A (2021). The effects of active video games on health-related physical fitness and motor competence in children and adolescents with healthy weight: a systematic review and meta-analysis. Int J Environ Res Public Health.

[69] Schmoigl-Tonis M, Schranz C, Müller-Putz GR (2023). Methods for motion artifact reduction in online brain-computer interface experiments: a systematic review. Front Hum Neurosci.

[70] Amjad I, Toor H, Niazi IK, Pervaiz S, Jochumsen M, Shafique M, Haavik H, Ahmed T (2019). Xbox 360 kinect cognitive games improve slowness, complexity of EEG, and cognitive functions in subjects with mild cognitive impairment: a randomized control trial. Games Health J.

[71] Schättin A, Baier C, Mai D, Klamroth-Marganska V, Herter-Aeberli I, de Bruin ED (2019). Effects of exergame training combined with omega-3 fatty acids on the elderly brain: a randomized double-blind placebo-controlled trial. BMC Geriatr.

[72] Jirayucharoensak S, Israsena P, Pan-ngum S, Hemrungrojn S, Maes M (2019). A game-based neurofeedback training system to enhance cognitive performance in healthy elderly subjects and in patients with amnestic mild cognitive impairment. CIA.

[73] Schättin A, Arner R, Gennaro F, de Bruin ED (2016). Adaptations of prefrontal brain activity, executive functions, and gait in healthy elderly following exergame and balance training: a randomized-controlled study. Front Aging Neurosci.

[74] Dimitrova J, Hogan M, Khader P, O'Hora D, Kilmartin L, Walsh JC, Roche R, Anderson-Hanley C (2017). Comparing the effects of an acute bout of physical exercise with an acute bout of interactive mental and physical exercise on electrophysiology and executive functioning in younger and older adults. Aging Clin Exp Res.

[75] Zhang H, Wang D, Wang Y, Chi Y, Miao C (2021). Development and validation of a practical instrument for evaluating players’ familiarity with exergames. International Journal of Human-Computer Studies.

[76] Dotson VM, Duarte A (2020). The importance of diversity in cognitive neuroscience. Ann N Y Acad Sci.

[77] Rico-Olarte C, Lopez D, Narváez S, Farinango CD, Pharow PS (2017). HapHop-Physio: a computer game to support cognitive therapies in children. PRBM.

[78] Rico-Olarte C, Lopez DM, Becker L, Eskofier B (2020). Towards classifying cognitive performance by sensing electrodermal activity in children with specific learning disorders. IEEE Access.

[79] (2023). Electrophysiological brain activity during a cognitive rehabilitation training for children with learning disorders. BayLat.

