# Supplementary material for: Electrophysiological Insights in Exergaming—Electroencephalography Data Recording and Movement Artifact Detection: Systematic Review
Source: JMIR Serious Games. 2025 Apr 7;13:e50992. doi: 10.2196/50992 (PMC12012405; doi:10.2196/50992)
Supplement: Multimedia Appendix 2 [file games_v13i1e50992_app2.pdf]

Table A. Filters and limits in information sources from search strings

**String search #1**	(**Exergaming** OR exer-gaming OR exergame OR exer-game OR "exerlearning game" OR "exer-learning game" OR "serious game" OR "interactive game" OR "cognitive-motor game" OR "motor-cognitive game" OR "motor-cognition game") **AND** (**EEG** OR electroencephalography OR electroencephalogram OR "brain activity" OR neurophysiology OR neuro-physiology OR ERP OR "event-related potential" OR "event related potential" OR "frequency spectrum" OR "gamma activity" OR gamma-activity OR "gamma band" OR gamma-band OR "gamma power" OR gamma-power OR "gamma frequency" OR gamma-frequency OR "alpha activity" OR alpha-activity OR "alpha band" OR alpha-band OR "alpha power" OR alpha-power OR "alpha frequency" OR alpha-frequency OR "theta activity" OR theta-activity OR "theta band" OR theta-band OR "theta power" OR theta-power OR "theta frequency" OR theta-frequency)		
**Source**	**String search applied in**	**Filters applied**	**Number of results and years**
PubMed/MEDLINE	Title/Abstract	**Include**<br>Article type:<br>Books and documents<br>Clinical trials<br>RCT	40<br>[2014-2024]
Web of Science	Title and Abstract	**Refine by**<br>Document types:<br>Article	50<br>[2011-2023]
IEEE Xplore	Document Title/Abstract	None	24<br>[2010-2023]
ACM DL	Title or Abstract or Keywords	**Include**<br>Content type:<br>Research article	101<br>[1997-2024]
Scopus	Title-Abstract-Keywords	**Include**<br>Document type:<br>Article<br>Book chapter<br>Publication stage:<br>Final<br>Language:<br>English	264<br>[2007-2024]
**String search #2** (Commercial exergames)	("**Oculus**" OR "Oculus Quest" OR "Meta Quest" OR "VR Headset" OR "PlayStationVR" OR "HTC Vive" OR "Nintendo Switch" OR "Kinect" OR "Xbox Kinect" OR "PlayStation Move" OR "Nintendo Wii") **AND** (**EEG** OR electroencephalography OR electroencephalogram OR "brain activity" OR neurophysiology OR neuro-physiology OR "cortical potential" OR "slow wave" OR slow-wave OR ERP OR "event-related potential" OR "event related potential" OR "frequency spectrum" OR "gamma activity" OR gamma-activity OR "gamma band" OR gamma-band OR "gamma power" OR gamma-power OR "gamma frequency" OR gamma-frequency OR "alpha activity" OR alpha-activity OR "alpha band" OR alpha-band OR "alpha power" OR alpha-power OR "alpha frequency" OR alpha-frequency OR "theta activity" OR theta-activity OR "theta band" OR theta-band OR "theta power" OR theta-power OR "theta frequency" OR theta-frequency)		
**Source**	**String search applied in**	**Filters applied**	**Number of results and years**
PubMed/MEDLINE	Title/Abstract	**Include**<br>Article type:<br>Books and documents	41<br>[2010-2024]

		Clinical trials<br>RCT	
Web of Science	Title and Abstract	**Refine by**<br>Document types:<br>Article	51<br>[2010-2023]
IEEE Xplore	Document Title/Abstract	None	51<br>[2012-2023]
ACM DL	Title and Abstract and Keywords	**Include**<br>Content type:<br>Research article	9<br>[2012-2022]
Scopus	Title-Abstract-Keywords	**Include**<br>Document type:<br>Article<br>Book chapter<br>Publication stage:<br>Final<br>Language:<br>English	558<br>[1957-2024]
