# Supplementary material for: Electrophysiological Insights in Exergaming—Electroencephalography Data Recording and Movement Artifact Detection: Systematic Review
Source: JMIR Serious Games. 2025 Apr 7;13:e50992. doi: 10.2196/50992 (PMC12012405; doi:10.2196/50992)
Supplement: Multimedia Appendix 3 [file games_v13i1e50992_app3.pdf]

Table B. Details of intervention or exposure in the studies.

Author	Sample size [n]	Age [Mean ± SD, years]	Exergame	Sessions [number]	Time [minutes per session]
[38] Anders, 2018	24<br>– 12 females<br>– 12 males	24,6 ± 2,1	"Puzzle": a sideways-leaning exergame with a 5-by-5 puzzle. Participants should lean in the correct left or right direction.	1	40
[39] Scherer, 2013	8<br>– 3 females<br>– 5 males	23	Kinect-based "catch the ball" game: participants are the goalkeepers, and they are tasked to touch the marked ball while seated in a chair.	1 (with 2 experimental runs)	30
[40] Ghani, 2021	24<br>– 11 females<br>– 13 males	25 ± 3,4	Tilt-ball game via a balance board. Participants move their center of mass to control the tilt direction and angle.	1 (with 6 separate runs)	60
[41] Parent, 2020	48	No report	(i) TIMEframe Puzzle game (non-stressful) (ii) Outlast Survival game (stressor). Movement while playing was induced by a static bike.	1 (with 2 runs)	60
[42] Xu, 2020	16<br>– 5 females<br>– 11 males	21,75	A VR exergame called GestureStar: the players are asked to make a body gesture to eliminate a block flying toward them.	1 (with 4 runs)	60
[43] Ko, 2020	10	28,5 ± 6,11	The exergame is a Ski Motion Platform. The scenario is an alpine ski racing with VR and No-VR conditions.	1 (with 2 experimental runs)	60
[44] Elor, 2022	5<br>– 1 female<br>– 4 males	24,5	Project Butterfly (PBF) is an exergame modified for upper limb rehabilitation. The flying pattern of the butterfly recreates the rehabilitation exercises.	16	50
[45] Baumeister, 2010	10 males	26 ± 0,7	Golf in Nintendo Wii	1 (with 2runs)	30
[46] Kandemir, 2021	10	24,5	(i) Fruit picking game: player collects falling fruits while moving a basket with their hands. (ii) Air hockey game: player moves the paddle with their hands to hit the puck or defend goals.	1	5

Author	Sample size [n]	Age<br>[Mean ± SD, years]	Exergame	Sessions [number]	Time [minutes per session]
[47] Pacheco, 2017	10<br>– 5 females<br>– 5 males	22,3 ± 2,75	A virtual game called "Basic step" for Nintendo Wii. The movement was ensured by the Wii Balance Board. The game makes the player move up and down.	1 (with two runs)	30
[48] Dang, 2017	5	9 ± 1,7	A story game based on Kinect	1	No report
[49] Fernandes, 2021	10<br>Right injury:<br>– 3 females<br>– 2 males<br>Left injury:<br>– 1 female<br>– 4 males	RI: 52,0 ± 10,93<br>LI: 48,8 ± 4,76	Kinect Sports game: virtual darts	12	60
[50] Olyaei, 2022	52<br>Exergame:<br>– 15 females<br>– 10 males<br>Control:<br>– 18 females<br>– 9 males	Exergame: 70.43 ± 5.24<br>Control: 71.22 ± 5.82	Wii Fit	16	60
[51] Müller, 2023	28<br>– 14 females<br>– 14 males	74.57 ± 0.78	SilverFit (balance training) with two exergames: puzzle and fox	1	45
[52] Romero-Borquez, 2023	31<br>– 18 females<br>– 13 males	25.7	Meta Quest 2 (VR headband) Exergame: Beat Saber	1	60
[53] Amprimo, 2023	50<br>– 13 females<br>– 37 males	26 ± 4.5	Grab-Drag-Drop (GDD) exergame	1	30

Author	Sample size [n]	Age<br>[Mean ± SD, years]	Exergame	Sessions<br>[number]	Time<br>[minutes per session]
[54]<br>Moinnereau, 2022	8<br>– 3 females<br>– 5 males	28.9 ± 2.9	HTC Vive (VR) exergame Half-Life: Alyx.	2	Session 1: 30<br>Session 2: 60
