# Supplementary material for: Electrophysiological Insights in Exergaming—Electroencephalography Data Recording and Movement Artifact Detection: Systematic Review
Source: JMIR Serious Games. 2025 Apr 7;13:e50992. doi: 10.2196/50992 (PMC12012405; doi:10.2196/50992)
Supplement: Multimedia Appendix 4 [file games_v13i1e50992_app4.pdf]

Table C. Details of EEG recordings in the studies.

Author	EEG device	EEG positions	Signal pre-processing	Outcome
[38] Anders, 2018	QuikCap Electrode System Analog amplifier: SynAmps (Scan 4.5)	64 Ag/AgCl passive electrodes according to the 10-20 system and a standard reference electrode positioned between CZ and CPZ.	Signal band limited between 1 Hz and 100 Hz. Signal down sampled to 250 Hz. CleanLine plugin [55] to remove line noise along with a finite impulse response filter with a band-pass between 2 Hz and 30 Hz. Non-stereotypical artifacts were removed by visual inspection. 47%–55% of the EEG data remained for further processing.	Central Alpha-2 power was significantly higher in exergame conditions compared to self-paced movement.
[39] Scherer, 2013	g.tec USBAmp	AFz, FC3, FCz, FC4, CP3, CPz, CP4, P1, P2, O1, and O2	Signal Quality Index [56] metric was applied to remove artifacts. Also, a band pass filter (third-order Butterworth filter) was used.	Alpha Event Related Desynchronization (ERD) during hand movement is stronger for the non-dominant hand.
[40] Ghani, 2021	EEG cap with a REFA amplifier (TMSi)	64 channels were recorded, N1 component was obtained from Fz, Cz, and Pz.	The PREP pipeline [57] was used to remove and interpolate bad channels, and to remove the line noise; the ICA method [58] was used to remove eye blinks and muscle artifacts. Then, data was band pass filtered at 0.05–30 Hz.	The mean value of the N1 component goes from low to high according to the difficulty level. For the easy level, the mean value is the lowest. For hard level, the mean value is higher.
[41] Parent, 2020	Muse headband	Prefrontal: AF7, AF8 Temporal-parietal: TP9, TP10	Automatic wavelet-enhanced ICA (wICA) for artifact removal was used.	Amplitude modulation rose during stress conditions. The apparent larger increase observed on temporal-parietal regions might have been caused by the greater influence of ocular and physical activity artifacts on prefrontal electrodes.
[42] Xu, 2020	Muse headband	Not reported	Authors do not detail how to process the signal or how to remove the motion artifacts.	The EEG measurements were used to get the engagement index (E) [Ref] based on the formula: $E = \beta / (\alpha + \theta)$.
[43] Ko, 2020	Brainno	Not reported	Artifacts detected in the EEG signal were removed using a finite impulse response filter.	The SMR (sensorimotor rhythm) wave from EEG is related to concentration. A higher concentration was achieved with the VR exercise condition than with non-VR exercise.
[44] Elor, 2022	InteraXon Muse 2	Prefrontal cortex: TP9, TP10, AF1, and AF7	Authors looked for movement generated. However, the EEG device helped them in the identification of eye blinks and jaw clenches.	Changes in all brainwave bands are noticeable between difficulty levels and goals.

Author	EEG device	EEG positions	Signal pre-processing	Outcome
[45] Baumeister, 2010	Electrocap Inc.	Frontal: Fz, F3, F4 Parietal: Pz, P3, P4 Central: Cz	The signal was high pass filtered at 0.86 Hz. Automatic artifact detection with respect to the EEG amplitude and slew rate was done followed by visual inspection.	Increased frontal theta activity due to higher attention was found in the real environment, as well as less cortical activity in the parietal Alpha-2 activity.
[46] Kandemir, 2021	Mindwave headset	Not reported	Authors do not detail how to process the signal or how to remove the motion artifacts.	Attention as obtained from the EEG software increased while playing physical activity-based games.
[47] Pacheco, 2017	Emotiv EPOC	Prefrontal: AF3, AF4 Frontal: F3, F4, F7, F8 Precentral: FC5, FC6 Occipital: O1, O2 Parietal: P7, P8 Temporal: T7, T8	EEG recording was cut into 10 seconds. A 1 Hz and 50 Hz band pass filter was applied along with visual analysis for the exclusion of noise.	Theta and alpha increase with a small to large size effect in real environments. In the virtual environment, beta, and gamma increase; small to negligible size effect for beta; medium to large size effect for gamma. Therefore, higher cognitive control is necessary to execute motor tasks in real environments.
[48] Dang, 2017	Mindwave headset	Single channel	Noise from the raw signals of EEG was removed by using the 1€ Filter [59], which uses a first order low-pass filter with an adaptive cut-off frequency. Filtered signals are segmented at a constant size by framing.	EEG features were manually labeled to build a classification model. An outcome based on the behavior of the signal was not reported.
[49] Fernandes, 2021	Emotiv EPOC	16 gold-plated contact sensors. Antero-frontal: AF3, AF4 Fronto-central: FC5, FC6 Temporal: T7, T8 Parietal: P7, P8	For obtaining the frequency-based analysis: band pass filter from DC (4 Hz) to 30 Hz and a Welch periodogram. For exclusion of noise caused by movement, the process is not well described.	RI: increase alpha and decrease beta. Changes presented in ipsilesional areas. Performance improved. LI: increase alpha no changes in beta. Changes in contralesional areas. Not improved performance.
[50] Olyaei, 2022	Brain Quick System	64-channels EEG electrodes of Cz, C3, and C4 were located to obtain late Contingent Negative Variation (CNV), alpha ERD, and beta ERD.	50 Hz notch filter was used to eliminate line noise.Signal epochs contaminated with artifacts were corrected by removing the artifact components. All epochs were visually inspected, and then uncorrected noisy trials through ICA were discarded.	A reduced late CNV and reduced alpha/beta ERD was found prior to gait initiation with a medium to large effect size.

Author	EEG device	EEG positions	Signal pre-processing	Outcome
[51] Müller, 2023	actiCAP and Live Amp 64	64 active EEG electrodes were applied with the ground electrode placed mid forehead and referenced online to FCz.	An EEG processing pipeline has been applied using the CleanLine plug-in to remove sinusoidal noise, the eBridge plugin to remove channels that were linked via electrical bridges due to low impedance. Additional noisy channels were removed with the EEGLAB pop_rejchan function. The clean_rawdata EEGLAB plugin was applied. Finally, large-amplitude artefacts were interpolated by applying automated subspace reconstruction (ASR).	Frontal theta power demonstrated a significant main effect of condition in both games. Alpha-2 power showed a significant main effect of condition in both the central and parietal areas.
[52] Romero-Borquez, 2023	MUSE 2	Not reported	Authors did not report the signal pre-processing step.	Strong focus was found on the gameplay of Beat Saber
[53] Amprimo, 2023	Dreem 2 Headband	6 EEG dry electrodes: signal was recorded at the frontal and pre-frontal sites, through channels Fpz, F7, F8 with occipital channels O1 and O2 set as reference.	Artifact rejection only encompassed pre-processing filters and very simple operations. Independent Component Analysis (ICA) was employed to remove ocular artefacts, using the Infomax algorithm [60].	Discrimination between rest and gaming states may be properly measured by means of a low-cost BCI. A binary classification between the REST and the GAME configuration was performed with the EEG features.
[54] Moinnereau, 2022	OpenBCI	11 EEG signals located: frontal area (Fp1, Fpz and Fp2), central area (F3, F4, FCz, C3 and C4), and occipital area (O1, Oz and O2)	Motion artifacts were removed by the artifact subspace reconstruction (ASR) method.	Six of eight participants showed a slightly higher positive engagement score during the exploration/combat condition. All participants showed low values for the arousal index and moderate values for the valence index, suggesting an overall positive emotion eliciting joy and happiness with a greater interest in the second condition. From the frontal alpha asymmetry (FAA) metric, most of the participants exhibited a negative value corroborating increased levels of fear.
