# Supplementary material for: Electrophysiological Insights in Exergaming—Electroencephalography Data Recording and Movement Artifact Detection: Systematic Review
Source: JMIR Serious Games. 2025 Apr 7;13:e50992. doi: 10.2196/50992 (PMC12012405; doi:10.2196/50992)
Supplement: Multimedia Appendix 5 [file games_v13i1e50992_app5.pdf]

Results of individual studies

Feasibility of Recording Brain Activity During Exergaming

According to its authors, [38] is the first study in demonstrating that it is feasible to record brain activity while exergaming. Cortical activity was measured with the EEG and the signal processing is well-detailed. The absolute power of the EEG signal was calculated as the area under the curve for each one of the clusters formed by the five resulting dipoles obtained from the adaptive mixture ICA decompositions. As result, a significantly higher power in the frontal cluster (theta θ band) was found during exergaming, as well as a decrease in the cortical activity of bilateral motor areas in the presence of cognitive load.

In [39], authors used the paradigm of functional brain mapping to have a comprehensive understanding of brain functions in the context of motor activity. Then, the authors used the concept of Kinect-based games and noninvasive EEG for mapping brain functions. The analysis of EEG signals was established by defining ERD and ERS maps. Since there was movement, the quality of the signal was ensured with the SQI metric to remove artifacts. The relative power of each band signal alpha (α) and beta (β) was obtained. Some of the results included sustained α-ERD patterns during hand movement, being stronger in the non-dominant hand.

Regarding [53], the paper explores the feasibility of using single-channel EEG for assessing mental workload and engagement during exergaming. A specially designed exergame (GDD) is tested on fifty healthy subjects, with questionnaires indicating high levels of engagement. Statistical analysis of EEG features reveals distinct patterns between rest and gameplay stages, indicating the potential for discriminating between these states. However, classification between different levels of gameplay based on EEG features yields overall poor results, possibly due to the combination of various stimuli and the short duration of some levels. Limitations of the study include recruitment of only healthy subjects and challenges related to electrode setup and recording quality.

Cognitive Workload and Rehabilitation Settings

Authors in [40] aimed to examine the efficacy of using ERPs of EEG signals as an outcome measure for cognitive workload in rehabilitation settings with exergames. They used the tilt-ball game which had eight goalposts; participants must move a soccer ball to each post by moving their center of mass. The authors stated that no objective measures of the cognitive workload associated with rehabilitation tasks have been taken before them. The analysis method consisted in obtaining the amplitude of the N1 component from the latency information of the grand-averaged ERP waveform during gaming. As a result, the amplitude of the N1 ERP component decreased significantly with an increase in task difficulty (scoring goals in posts with obstacles).

In [44], authors explored the effects of an immersive VR rehabilitation exergame for upper limbs. Physical and biometric signals, including EEG, were recorded over the

two-month intervention. The intervention had two phases: a foundation part where users emphasize recovery range of motion and strength, and a challenge part where users pushed a range of motion recovery with higher weights. Regarding the EEG measurement, differences between difficulty levels and goals were analyzed and noticeable in the brain waves. Authors related five events to each band wave: stress for α, focus with β, awareness with delta (δ), motor with Θ, and general cognition with gamma (γ). The α band power indicated that challenge protocol has greater difficulty than the foundation protocol. The β wave spiked higher when difficulty was increased (more focus needed). Playing the exergame enabled to elicit responses for upper limb rehabilitation compared to traditional therapy.

Study in [50] explored the effects of exergaming on cognition and brain activity in older adults, focusing on motor-related cortical potentials (MRCPs) as indicators of preparatory brain activity. Participants engaged in exergaming sessions designed to stimulate motor and cognitive functions, while MRCPs were recorded to assess brain activity. The results revealed reductions in MRCP amplitude and alpha/beta event-related desynchronization (ERD) during exergaming, indicating decreased cortical excitability and improved neural efficiency. Additionally, both exergaming and traditional motor-cognitive training led to improvements in inhibitory control and information processing speed, with exergaming showing greater enhancements in cognitive flexibility. Authors stated that with these findings, exergaming offers a promising approach for enhancing cognitive function in older adults, potentially contributing to more effective rehabilitation programs in clinical settings.

Also, the study in [51] investigated the impact of games characteristics on brain activity and physical activity in older adults during exergaming sessions. Utilizing two exergames, a puzzle game and a fox game, participants' physical movement and brain activity were monitored. Results indicated that physical activity decreased with increased difficulty levels in both games, despite participants reporting higher perceived exertion in the puzzle game. Concurrently, increased frontal theta activity was observed across both games and difficulty levels, indicating heightened cognitive demands during exergaming. However, alpha-2 power displayed inconsistent patterns, suggesting potentially task-specific cognitive processing. According to the authors, their findings emphasize the importance of tailored exergaming interventions and highlight frontal theta activity as a potential biomarker for cognitive processing.

Stress Modulation and Affective Responses

In [41], a multimodal dataset of Physical Activity and Stress was introduced. Physiological signals such as EEG, electrocardiogram, electrodermal activity, breathing, body temperature, and blood volume pulse were collected during interaction with two different games. These games served to modulate the induction of affective stress (and mental stress differentiation) in players. In addition, the players varied their level of physical activity (three levels depending on the speed) by playing while pedaling on a stationary bike. For the EEG signal, wavelet-enhanced ICA methods were used for artifact removal. From the signal, average values and the mean

confidence interval of the EEG-selected features (absolute power, relative power, asymmetry, coherence, and amplitude modulation) were obtained. The paper presented a detailed dataset to monitor stress detection through models considering the measurement of these signals in the wild. Due to this condition, physical activity was introduced to identify affective stress from mental stress through physiological measurements that are susceptible to motion artifacts.

Two complementary studies were reported in [42]. The first study aimed at the identification of full-body gestures to be incorporated into the exergame. In the second study, participants played under combinations of different conditions of the VR exergame. The authors used EEG recordings with the exergame for only seven minutes. These signals were used to get the engagement index to confirm that the display type and the viewing perspective in the exergame do not affect the index. This engagement measure was obtained from the average values of α, β, and Θ bands. Playing with an exergame that requires full-body gestures led to a higher exertion level and a lower negative feeling in a VR environment. The engagement index was not affected by the evaluated conditions.

VR Effects on Cognitive Function and Concentration

Authors in [43] used a ski simulator to report brain activity collected by EEG based on the premise that physical activity generated by exergames along with the cognitive function is enhanced by interacting with an exergame. The objective of the study was to determine the effects of using VR in exergames. The weight of each EEG component was obtained through power spectrum analysis in the frequency domain. Particularly, the sensorimotor rhythm waves determined the state of concentration. A significant difference was found between the VR and non-VR conditions, indicating that VR exergame improved concentration.

The aim of [47] is similar to that described in [45]. However, the authors only compare the behavior of the band frequencies as well as the size effect. The comparison is made in adults performing a lower limb motor task in a real and a virtual environment. The virtual environment consists of a Nintendo Wii game called "Basic step" with the Balance Board. EEG is measured during just one minute. The experiment throws the same results as in the other study. Given the size effects of the Θ power, cognitive control is necessary to execute motor tasks in real environments. Although, Θ and α were higher in the virtual environment.

In [54], authors presented a novel protocol for remotely collecting physiological data during home-based VR studies with minimal intervention. By equipping a VR headset with sensors, data was gathered from participants' homes, even amidst COVID-19 lockdowns. Analysis revealed correlations between bio-signal metrics and key Human Influential Factors (HIFs) such as emotion, engagement, and immersion. Physiological signals, including ECG and EEG, showed strong correlations with subjective ratings, indicating their potential as objective measures of user experience.

In the same line of action, the study in [52] investigated the impact of VR video games on cognitive concentration levels, focusing on three genres: Challenging Puzzlers, Casual Games, and Exergames. With an increasing interest in the potential benefits of video games for mental health, particularly in managing emotions and enhancing concentration, the research aimed to understand how different video game genres affect concentration. Through EEG data analysis, the study measures power spectral density (PSD) in the alpha band, individual alpha frequency (IAF), and frontal alpha asymmetry (FAA) to assess concentration levels. The results indicated that exergames ("Beat Saber") elicit the highest level of concentration among players. The authors hope for future research to explore optimal usage patterns and individual factors influencing the effectiveness of video games in enhancing cognitive performance.

Attention and Cognitive Skills Improvement

In [46], two different physical activity-based games were developed and implemented. The final aim is to target deaf children by helping them to improve their attention, emotions, and sensory-motor coordination. However, this study is just the first step. The subjects for experiments were adults. EEG was measured to obtain attention levels while participants were idle and while playing with the exergames. These games are based on the Kinect sensor. The aim of the study is to show that playing physical activity-based games supports the improvement of cognitive skills. The authors rely on the metrics obtained from the software and no signal processing was reported in the paper. Results of the experiment showed that in 70% of the participants, the difference was significant in the attention between the idle position and the playing state.

The purpose of the study by [49] was to evaluate two measures of motor learning in individuals who have suffered from a stroke. The first measure was performance and the second was brain activity verified through EEG signals. Additionally, to this purpose, the results that can be obtained depend on the laterality of the lesion in a VR training environment. The results obtained supported the author’s hypothesis that there are differences in the rehabilitation processes that must be considered to have an effect, depending on the laterality of the injury. The performance of motor learning was measured as the decrease in the absolute error of the values of the dart-throwing game.

Motion Artifact Detection and EEG Signal Processing

In [48], the authors presented the development of a classifier that identifies if the child is doing certain movements given by the game, and if the child is engaged with the game or not. The aim of this paper was to explore the best evaluation for the classifier. To collect the data, it was assumed that the EEG data could be recorded during the gaming session even in the presence of movements. There is no description of the type of game, only the type of movement. The notation about the child's engagement was made based on observations of teachers who helped in the labeling process of the data.

Authors in [45] aimed at demonstrating that there is a difference in cortical activity patterns related to the working memory during a golf putting task in a real and a virtual environment using the Nintendo Wii. Ten volunteers with experience in playing golf were instructed to putt in a real environment vs. the Nintendo Wii golf game. The cortical activation pattern demonstrated differences in frontal Θ power and α-2 spectral values when comparing a putting performance in a real and a virtual environment using the Nintendo Wii. The frontal Θ power was able to discriminate between performance and rest.
