# Supplementary material for: Electrophysiological Insights in Exergaming—Electroencephalography Data Recording and Movement Artifact Detection: Systematic Review
Source: JMIR Serious Games. 2025 Apr 7;13:e50992. doi: 10.2196/50992 (PMC12012405; doi:10.2196/50992)
Supplement: Multimedia Appendix 6 [file games_v13i1e50992_app6.pdf]

**Quality assessment for papers**

| No | Observational cohort and Cross-sectional studies | Importance of question | Paper 1. Exergames inherently contain cognitive elements as indicated by cortical processing | | | Paper 2. On the use of games for noninvasive EEG-based functional brain mapping | | | Paper 3. Efficacy of a Single-Task ERP Measure to Evaluate Cognitive Workload During a Novel Exergame | | | Paper 4. PASS: A Multimodal Database of Physical Activity and Stress for Mobile Passive Body/Brain-Computer Interface Research | | | Paper 5. Studying the Effect of Display Type and Viewing Perspective on User Experience in Virtual Reality Exergames | | | Paper 6. Effects of Virtual Reality and Non–Virtual Reality Exercises on the Exercise Capacity and Concentration of Users in a Ski Exergame: Comparative Study | | | Paper 7. Gaming Beyond the Novelty Effect of Immersive Virtual Reality for Physical Rehabilitation | | | Paper 8. Brain activity in goal-directed movements in a real compared to a virtual environment using the Nintendo Wii | | | Paper 9. Development of adaptive human–computer interaction games to evaluate attention | | |
|---|---|---|---|---|---|---|---|---|---|---|---|---|---|---|---|---|---|---|---|---|---|---|---|---|---|---|---|---|---|
| | | | Yes | No | CD; NA; NR* | Yes | No | CD; NA; NR | Yes | No | CD; NA; NR | Yes | No | CD; NA; NR | Yes | No | CD; NA; NR | Yes | No | CD; NA; NR | Yes | No | CD; NA; NR | Yes | No | CD; NA; NR | Yes | No | CD; NA; NR |
| 1 | Was the research question or objective in this paper clearly stated? | Very important: if answer is NO, it would be immediately rated as poor quality | | | | | | | | | | | | | | | | | | | | | | | | | | | |
| 2 | Was the study population clearly specified and defined? | Very important: if answer is NO, it would be immediately rated as poor quality | | | | | | | | | | | | | | | | | | | | | | | | | | | |
| 3 | Was the participation rate of eligible persons at least 50%? | Less important | | | NA | | | NA | | | NA | | | NA | | | NA | | | NA | | | NA | | | NA | | | NA |
| 4 | Were all the subjects selected or recruited from the same or similar populations (including the same time period)? Were inclusion and exclusion criteria for being in the study prespecified and applied uniformly to all participants? | Important | | | | | | | | | | | | | | | | | | | | | | | | | | | |
| 5 | Was a sample size justification, power description, or variance and effect estimates provided? | Less important | | | | | | | | | | | | | | | | | | | | | | | | | | | |
| 6 | For the analyses in this paper, were the exposure(s) of interest measured prior to the outcome(s) being measured? | Less important | | | | | | | | | | | | | | | | | | | | | | | | | | | |
| 7 | Was the timeframe sufficient so that one could reasonably expect to see an association between exposure and outcome if it existed? | Less important | | | | | | | | | | | | | | | | | | | | | | | | | | | |
| 8 | For exposures that can vary in amount or level, did the study examine different levels of the exposure as related to the outcome (e. g., categories of exposure, or exposure measured as continuous variable)? | Less important | | | | | | | | | | | | | | | | | | | | | | | | | | | |
| 9 | Were the exposure measures (independent variables) clearly defined, valid, reliable, and implemented consistently across all study participants? | Very important: if answer is NO, it would be immediately rated as poor quality | | | | | | | | | | | | | | | | | | | | | | | | | | | |
| 10 | Was the exposure(s) assessed more than once over time? | Less important | | | | | | | | | | | | | | | | | | | | | | | | | | | |
| 11 | Were the outcome measures (dependent variables) clearly defined, valid, reliable, and implemented consistently across all study participants? | Very important: if answer is NO, it would be immediately rated as poor quality | | | | | | | | | | | | | | | | | | | | | | | | | | | |
| 12 | Were the outcome assessors blinded to the exposure status of participants? | Less important | | | NR | | | NR | | | NR | | | NR | | | NR | | | NR | | | NR | | | NR | | | NR |
| 13 | Was loss to follow-up after baseline 20% or less? | Less important | | | NA | | | NA | | | NA | | | NA | | | NA | | | NA | | | | | | NA | | | NA |
| 14 | Were key potential confounding variables measured and adjusted statistically for their impact on the relationship between exposure(s) and outcome(s)? | Less important | | | | | | | | | | | | | | | NR | | | | | | | | | | | | |

* CD: cannot determine; NA: not applicable; NR: not reported

| | | | | | | | | |
|---|---|---|---|---|---|---|---|---|
| FAIR | FAIR | GOOD | POOR | POOR | POOR | POOR | FAIR | POOR |

**Quality assessment for papers**

| No | Observational cohort and Cross-sectional studies | Importance of question | Paper 10. Brain activity during a lower limb functional task in a real and virtual environment: A comparative study | | | Paper 11. An efficient movement and mental classification for children with autism based on motion and EEG features | | | Paper p12. Is motor learning of stroke patients in non-immersive virtual environment influenced by laterality of injury? A preliminary study | | | Paper p13. The effect of exergaming on cognition and brain activity in older adults: A motor- related cortical potential study | | | Paper p14. Exergaming in older adults: the effects of game characteristics on brain activity and physical activity | | | Paper p15. Mapping EEG Alpha Activity: Assessing Concentration Levels during Player Experience in Virtual Reality Video Games | | | Paper p16. Measuring Brain Activation Patterns from Raw Single-Channel EEG during Exergaming: A Pilot Study | | | Paper p17. Instrumenting a virtual reality headset for at-home gamer experience monitoring and behavioural assessment | | |
|---|---|---|---|---|---|---|---|---|---|---|---|---|---|---|---|---|---|---|---|---|---|---|---|---|---|---|
| | | | Yes | No | CD; NA; NR | Yes | No | CD; NA; NR | Yes | No | CD; NA; NR | Yes | No | CD; NA; NR | Yes | No | CD; NA; NR | Yes | No | CD; NA; NR | Yes | No | CD; NA; NR | Yes | No | CD; NA; NR |
| 1 | Was the research question or objective in this paper clearly stated? | Very important: if answer is NO, it would be inmediately rated as poor quality | | | | | | | | | | | | | | | | | | | | | | | | | |
| 2 | Was the study population clearly specified and defined? | Very important: if answer is NO, it would be inmediately rated as poor quality | | | | | | | | | | | | | | | | | | | | | | | | | |
| 3 | Was the participation rate of eligible persons at least 50%? | Less important | | | NA | | | | | | NA | | | | | | NA | | | NA | | | NA | | | NA |
| 4 | Were all the subjects selected or recruited from the same or similar populations (including the same time period)? Were inclusion and exclusion criteria for being in the study prespecified and applied uniformly to all participants? | Important | | | | | | NR | | | | | | | | | | | | | | | | | | NR |
| 5 | Was a sample size justification, power description, or variance and effect estimates provided? | Less important | | | | | | | | | | | | | | | | | | | | | | | | | |
| 6 | For the analyses in this paper, were the exposure(s) of interest measured prior to the outcome(s) being measured? | Less important | | | | | | | | | | | | | | | | | | | | | | | | | |
| 7 | Was the timeframe sufficient so that one could reasonably expect to see an association between exposure and outcome if it existed? | Less important | | | | | | | | | | | | | | | | | | | | | | | | | |
| 8 | For exposures that can vary in amount or level, did the study examine different levels of the exposure as related to the outcome (e. g., categories of exposure, or exposure measured as continuous variable)? | Less important | | | | | | | | | NA | | | | | | | | | | | | | | | | |
| 9 | Were the exposure measures (independent variables) clearly defined, valid, reliable, and implemented consistently across all study participants? | Very important: if answer is NO, it would be inmediately rated as poor quality | | | | | | | | | | | | | | | | | | | | | | | | | |
| 10 | Was the exposure(s) assessed more than once over time? | Less important | | | | | | | | | | | | | | | | | | | | | | | | | |
| 11 | Were the outcome measures (dependent variables) clearly defined, valid, reliable, and implemented consistently across all study participants? | Very important: if answer is NO, it would be inmediately rated as poor quality | | | | | | | | | | | | | | | | | | | | | | | | | |
| 12 | Were the outcome assessors blinded to the exposure status of participants? | Less important | | | NR | | | NR | | | NR | | | | | | NR | | | NR | | | NR | | | NR |
| 13 | Was loss to follow-up after baseline 20% or less? | Less important | | | NA | | | NA | | | | | | NA | | | NA | | | NA | | | NA | | | NA |
| 14 | Were key potential confounding variables measured and adjusted statistically for their impact on the relationship between exposure(s) and outcome(s)? | Less important | | | | | | | | | | | | | | | | | | | | | | | | | |
| * CD: cannot determine; NA: not applicable; NR: not reported | | | FAIR | | | POOR | | | FAIR | | | GOOD | | | FAIR | | | POOR | | | FAIR | | | POOR | | |
